# Trend analysis of leprosy in Morocco between 2000 and 2017: Evidence on the single dose rifampicin chemoprophylaxis

**DOI:** 10.1371/journal.pntd.0006910

**Published:** 2018-12-20

**Authors:** Ibtissam Khoudri, Zainab Elyoussfi, Yassine Mourchid, Mohammed Youbi, Nada Bennani Mechita, Redouane Abouqal, Abderrahmane Maaroufi

**Affiliations:** 1 Directorate of Epidemiology and Diseases Control, Ministry of Health, Rabat, Morocco; 2 Laboratory of Biostatistics Clinical Research and Epidemiology, Faculty of Medicine and Pharmacy-Mohamed V University, Rabat, Morocco; 3 Laboratory of Public Health, Faculty of Medicine and Pharmacy-Mohamed V Universit, Rabat, Morocco; University of Tennessee, UNITED STATES

## Abstract

**Background:**

Morocco has achieved the goal of leprosy elimination as a public health problem several years ago (less than 1 case/ 10 000 habitant). The aim of this study was to analyze trends of leprosy detection during the last 17 years taking into consideration the implementation of single dose rifampicin chemoprophylaxis (SDRC) started in 2012.

**Methodology:**

Time series of leprosy cases detected at national level between 2000 and 2017. Variable collected for each year were leprosy per 100000 H, age category, gender, origin, regions, grade of disabilities and clinical forms. The detection time series was assessed by Joinpoint Regression Analysis. Annual percentage changes (APCs) were estimated to identify the years (joinpoint) when significant changes occurred in the trend. We therefore examined trends in leprosy detection according to epidemiological variables.

**Findings:**

Joinpoint regression showed a reduction in the detection rate between 2000 and 2017. The APC for the period 2012–2017 (-16.83, 95% CI: -29.2 to -2.3, p <0.05) was more pronounced than that of the previous period 2000–2012 (- 4.68, 95% CI: -7.3 to -2.0, p <0.05); with a significant break in the same joinpoint year SDRC implementation. In stratified analysis, case detection decreased, but not significantly, after the joinpoint years in men, children, multi-bacillary cases, grade 0–1 disabilities, rural and urban cases and in ten regions.

**Conclusions:**

Leprosy detection was declining over years with a significant reduction by 16% per year from 2012 to 2017. SDRC may reduce leprosy detection over the years following its administration.

## Author summary

After the introduction of effective therapy for leprosy by the world health organization in early eighties, there has been a marked reduction in new case detection all over the world. Despite the decline in case detection of leprosy, incidence reduction to zero and suppression of infection source is still not possible. Addressed strategies to high-risk groups–contacts of leprosy cases- may be effective to eliminate the disease at global level. Chemoprophylaxis in contacts may lower the incidence of leprosy in contacts of patients diagnosed with leprosy. In Morocco, chemoprophylaxis was introduced at national level in 2012. Morocco has achieved the goal of leprosy elimination as a public health problem in 1991 and the declining trend of leprosy detection is observed years after years. However, no study has been conducted in the country to analyze whether the declining trend of leprosy can be related or not to chemoprophylaxis in contact. This time series can be of interest for medical community as the authors analyses the trend in leprosy in the last 17 years and try to generate a hypothesis about any relationship with chemoprophylaxis implementation.

## Introduction

Leprosy is a chronic infectious disease known since antiquity that mainly damages the skin and peripheral nerves. Leprosy can lead to severe forms of disability and definitive sequelae. Global control actions for leprosy have been a worldwide success since the introduction of antibacterial multidrug therapy (MDT) in 1981. Indeed, global number of leprosy cases has decreased from 5.4 million in the early 1980s to 210 758 in 2015 [1].

Despite this progress, leprosy control actions based on a secondary detection strategy can not lead to leprosy elimination nor suppress the source of infection [2]. Additional control intervention toward high-risk groups is then needed to interrupt transmission. Household contacts of leprosy cases are considered the main source of infection [3–4]. Single dose Rifampicin Chemoprophylaxis (SDRC) of leprosy contacts is a preventive intervention that may stop the transmission of infection among household contacts [5]. A randomized controlled trial [6] including more than 28 000 contacts in Bangladesh showed evidence that SDRC reduces the incidence of leprosy by 57% in the first two years following the intervention. This effect is maintained after 4 to 6 years. A meta-analysis [2] confirmed also the effectiveness of SDRC in preventing new leprosy cases after two years.

In Morocco, great efforts were undertaken over several decades to control leprosy. The country achieved the goal of leprosy elimination as a public health problem in 1991 [7] and the decreasing trend in new case detection is confirmed year by year. SDRC was introduced in Morocco in 2012 among household contacts of accumulated leprosy cases registered in the country since 2000 as well as the household contacts of new cases registered from 2012. To our knowledge, this is the first time series on leprosy conducted in a country that adopted SDRC in its interventions and at a national level.

The aim of this study was to examine the trends of new leprosy cases detected between 2000 and 2017, as well as to generate a hypothesis suggesting any link between the decrease in the number of detected cases and the introduction of SDRC in Morocco.

## Methods

### Study design

We performed a time series study based on new leprosy cases data registered in Morocco between 2000 and 2017. Morocco is a northwestern African country that includes 12 administrative districts or regions and more than 70 provinces. The ethical committee of the Faculty of Medicine and Pharmacy of Rabat approved the study protocol. All data analyzed were anonymized.

### Data collection

Leprosy detection rate per 100 000 habitant was calculated from the data of the National Leprosy Control Program (NLCP) and the population data obtained from *le Haut Commissariat au Plan* for the period 2000–2017. For each year, the detection rate was the number of new leprosy cases registered in the country reported per 100 000 habitant [7]. Data collected, for each year, were also age category (adult, child), gender, origin (rural, urban), grade of disability, regions and clinical leprosy forms (multi-bacillary or pauci-bacillary) of all the new cases registered during the same period.

Data from SDRC surveys among household contacts of cumulative leprosy cases registered from 2000 to 2017 were also collected. Household contacts of a leprosy case index were defined as people living under the same roof with the case index for more than three months [8]. SDRC was administered during the leprosy case investigations conducted by regional NLCP teams. SDRC was administered after a counseling session and a careful clinical examination of contacts in order to eliminate contraindications to rifampicin [8]. SDRC was administered to contacts with respect for ethics and confidentiality.

### Statistical analysis

First, the observed trend of leprosy detection rate was described between 2000 and 2017. Then, a Joinpoint Regression Analysis was conducted to assess the time trends [9]. Joinpoint regression detects years when a significant change in leprosy detection trend has occurred. This method is widely used in trend analysis of incidence rate or mortality of several diseases [10].

Joinpoint regression analysis was adjusted to estimate the Annual Percent Changes (APCs) and to identify the joinpoints or years where significant changes over time in the linear slope of the trend had occurred. Statistical significance was tested using the Monte Carlo Permutation method [9]. A maximum of one joinpoint was allowed in the model and each APC segment was calculated using a log-linear model. The 95% confidence intervals (95% CI) were calculated for each APC and were used to determine whether the APC for each segment was significantly different from the previous time segment. A *p* value <0.05 was considered statistically significant. Moreover, a stratified analysis by age (adult child), gender, clinical forms (multibacillary, paucibacillary), origin (rural, urban), regions and disability grades were also studied. Joinpoint regression analysis was performed using Joinpoint Regression Program, version 4.2.0.2 (United states National Cancer Institute, Bethesda, MD, USA) [11].

## Results

### Epidemiological characteristics of leprosy between 2000–2017

A total of 801 new leprosy cases were recorded between 2000 and 2017. Epidemiological characteristics of registered cases are shown in Table 1. The number of new cases detected decreased from 61 cases in 2000 to 13 cases in 2017. Leprosy detection rate was declining since the early 2000s, as the annual detection rate decreased from 0.21 per 100 000 H in 2000 to 0.04 per 100 000 H in 2017.

**Table 1:** Epidemiological characteristics of leprosy in Morocco during the period 2000–2017.

	n (%)
New cases*	44±16
Leprosy detection rate/ 100 000 H*	0.14±5.5 10−^2^
**Gender**	
• Male	509 (64)
• Female	292 (36)
**Age**	
• Adult	749 (94)
• Child	52 (6)
**Leprosy clinical forms**	
• Multi-bacillary	(65)
• Pauci-bacillary	282 (35)
**Origin**	
• Urban	(27)
• Rural	580 (73)
**Disability grade**	
• 0–1	(90)
• 2	78 (10)
**Regions**	
• Tanger Tétouan Al Hoceima	(28)
• Oriental	(8)
• Fès-Meknès	(23)
• Rabat-Salé-Kénitra	(15)
• Béni-Mellal-Khénifra	(5)
• Casablanca-Settat	(11)
• Marrakech-Safi	(5)
• Drâa-Tafilalet	(1)
• Sous-Massa	(2)
• Guelmim-Oued Noun	(1)
• Laâyoune-Sakia- Al Hamra	3 (0.4)
• Dakhla-Oued Ed-Dahab	5 (0.6)

* mean ± SD.

### Single dose rifampicin chemoprophylaxis

**Fig 1:**
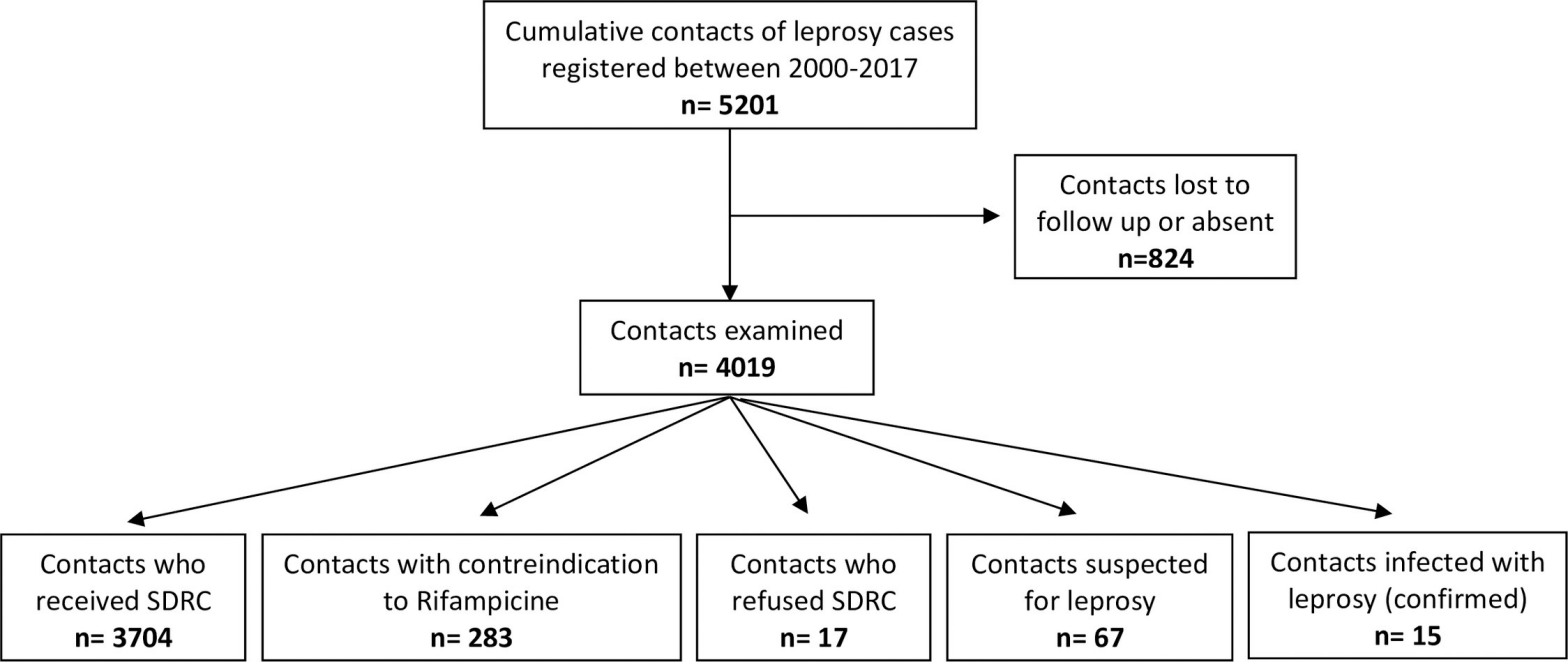
Flow chart of contact investigations and implementation of single dose rifampicin chemoprophylaxis.

Since the introduction of SDRC into the routine activities of the NLCP in 2012, 146 investigations were conducted by the regional teams. Among 5201 household contacts registered, 4019 (77%) contacts were examined during investigations, of whom 3704 (93%) received SDRC (Fig 1).

### Time series of leprosy detection

**Fig 2:**
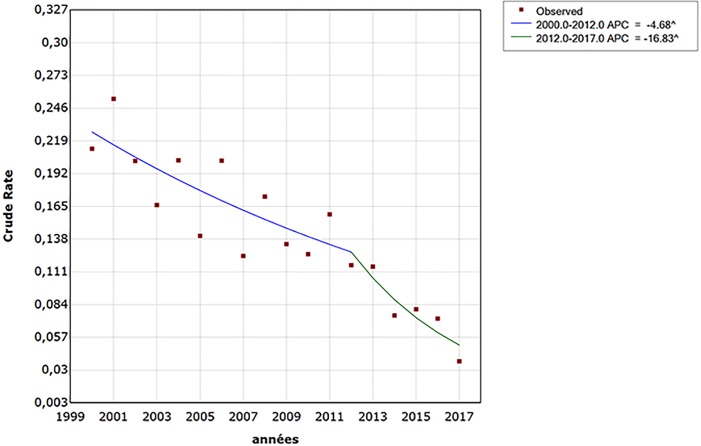
Observed and analyzed trend of leprosy detection in Morocco between 2000 and 2017. APC: Annual percent change. ^difference statistically significant (p<0.05).

The joinpoint regression analysis showed a significant reduction in leprosy detection between 2000 and 2017 (Fig 2). The leprosy detection rate decreased by 4% per year from 2000 to 2012 and by 16% from 2012 to 2017. The APC for the period 2012–2017 (APC = -16.83, 95% CI: - 29.2 to -2.3, p <0.05) was then more pronounced than the previous period 2000–2012 (APC = -4.68, 95% CI: -7.3 to -2.0; <0.05). The joinpoint corresponded to the year 2012.

**Fig 3:**
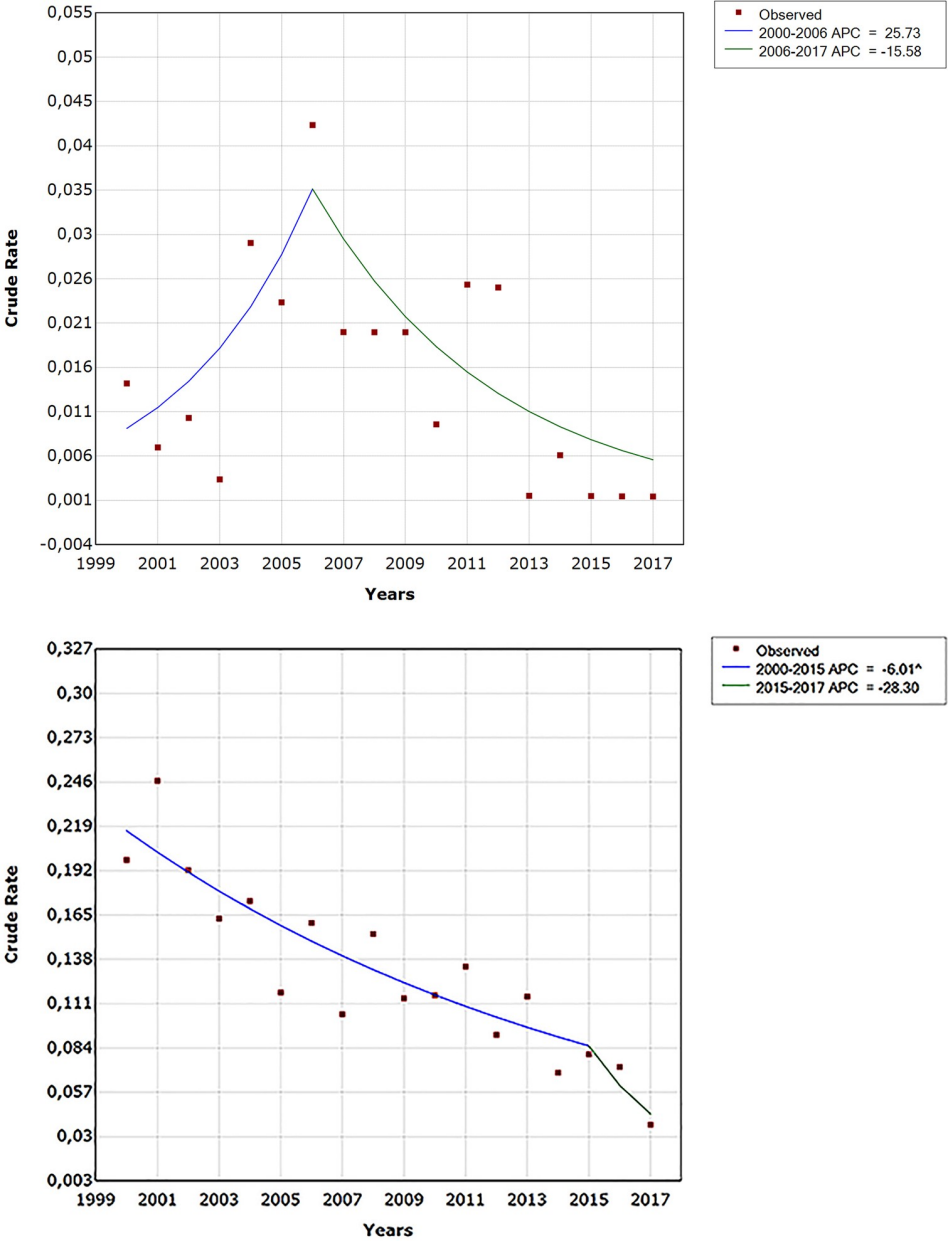
Joinpoint regression for leprosy detection by disabilities during the period 2000–2017.

The stratified joinpoint regression analysis (Table 2) showed that the detection rate decreased significantly after the joinpoint years in female cases (APC = -12.3, 95% CI: -18.1 to -6.3; <0.05), adults (APC = -17.0, 95% CI: -29.7 to -2.0, p <0.05), paucibacillary forms (APC = -25.0, 95% CI: -37.1 to -10.8, p <0.05) and in two regions: Béni-Mellal-Khenifra (APC = -22.9, 95% CI: -35.2 to -8.1, p<0.05) and Marrakech-Safi (APC = -8.9, 95% CI: -16.3 to -0.8, p<0.05). The detection rate decreased also but not significantly after the joinpoint years in men, children, multibacillary cases, grade 0–1 disability cases and rural and urban cases. For grade 2 disability cases, the detection rate increased insignificantly from 2000 to 2006 in (APC = 25.7, 95% CI: -5.4 to 67.1) followed by a non-significant decrease in the second period (Fig 3).

**Table 2:** Stratified joinpoint regression analysis for leprosy detection during the period 2000–2017.

	*Period from 2000 to the year joinpoint*			*Joint-point* (year)	*Period from the joinpoint to 2017*		
	APC	CI 95%	*p*		APC	CI 95%	*p*
**Gender**							
• Male	-4.9	-7.7 to -2.1	<0.05	2013	-19.6	-40.1 to 7.7	NS
• Female	-2.3	-7.8 to 3.4	NS	2008	-12.3	-18.1 to -6.3	<0.05
**Age**							
• Adult	-4.5	-7.4 to -1.6	<0.05	2012	-17.0	-29.7 to -2.0	<0.05
• Child	-10.8	-15.7 to -5.7	<0.05	2014	-35.4	-84.7 to 175.6	NS
**Clinical forms**							
• MB	-4.9	-8.6 to -1.1	<0.05	2015	-29.8	-83.6 to 199.8	NS
• PB	1.9	-5.3 to 9.8	NS	2009	-25.0	-37.1 to -10.8	<0.05
**Disabilities**							
• Grade 0–1	-6.0	-8.0 to -3.9	<0.05	2015	-28.3	-68.5 to 63.4	NS
• Grade 2	25.7	-5.4 to 67.1	NS	2006	-15.5	-29.3 to 1.8	NS
**Origin**							
• Urban	-1.8	-5.6 to 2.1	NS	2014	-24.6	-55.8 to 28.4	NS
• Rural **Regions** • Tanger Tétouan Al Hoceima • Oriental • Fès-Meknès • Rabat-Salé-Kénitra • Béni-Mellal-Khénifra • Casablanca-Settat • Marrakech-Safi • Drâa-Tafilalet • Sous-Massa • Guelmim-Oued Noun • Laâyoune-Sakia- Al Hamra • Dakhla-Oued Ed-Dahab **Total leprosy cases**	-6.0 -1.8 -7.0 -2.6 -8.0 -4.4 -1.9 18.4 -32.1 8.4 7.1 -7.9 7.4 -4.6	-9.0 to -2.9 -6.1 to 2.7 -16.0 to 2.9 -10.8 to 6.4 -11.4 to -4.5 -30.4 to 31.2 -8.5 to 5.1 -61.4 to 263.5 -60.2 to 15.7 0.1 to 17.3 -5.2 to 21.0 -31.5 to 23.7 -1.5 to 17.1 -7.3 to -2.0	<0.05 NS NS NS <0.05 NS NS NS NS <0.05 NS NS NS <0.05	2012 2013 2012 2011 2015 2006 2014 2006 2006 2014 2008 2006 2012 2012	-15.5 -41.0 4.1 -6.3 -22.8 -22.9 -53.3 -8.9 -2.9 -47.1 -5.2 -3.3 -24.3 -16.8	-30.9 to 3.2 -65.5 to 0.9 -28.8 to 52.2 -30.3 to 26.1 -83.6 to 264.2 -35.2 to -8.1 -90.4 to 127.2 -16.3 to -0.8 -16.1 to 12.4 -80.4 to 43.2 -14.8 to 5.5 -8.0 to 1.5 -50.0 to 14.6 -29.2 to -2.3	NS NS NS NS NS <0.05 NS <0.05 NS NS NS NS NS <0.05

APC: Annual percent changes; CI: Confidance interval; MB: Multibacillary forms; PB: Paucibacillary forms; NS: Différence statistically non significative (p>0.05)

APC: Annual percent change. ^difference statistically significant (p<0.05).

## Discussion

This study shows that leprosy detection was declining over years with a significant reduction by 16% since the introduction of SDRC in Morocco. Leprosy control strategies in the country were implemented through NLPC. NLCP is one of the oldest programs in the Ministry of Health; it was set up in 1981. Control actions for leprosy in Morocco started since the fifties by the establishment of a prophylactic and socio-medical policy. Today, NLCP achievements are evident since the epidemiological situation of leprosy confirms the decreasing trend in new case detection. An average of 26 new leprosy cases was detected during the last five years. NLCP control actions have given the opportunity to many patients, mostly of low socioeconomic level, to be treated free of charge and, if necessary, rehabilitated for a better reintegration into society.

Leprosy in Morocco remains contagious and the genetic predisposition to this disease explain the fact that more than 50% of new cases diagnosed have either a leprosy family contact and more than 60% are from four high endemicity aeras for leprosy [8]. Given that, NLCP has accorded a close attention to contact surveillance.

Several publications have demonstrated SDRC cost-effectiveness among contacts with 50–60% efficiency [12–14]. However, there is no recommendation of World Health Organization (WHO) to implement SDRC in low or high endemic countries because of low quality of evidence in the field. WHO experts in leprosy advise low endemic countries, such as Morocco, to implement SDRC into routine program activities since they already have a leprosy surveillance system in place. [1]. SDRC was then implemented at national level in Morocco among household contacts with the aim to reduce leprosy transmission among contacts.

Our study showed that leprosy detection has been declining since 2000 with a significant break in the curve occurring in 2012. Leprosy detection has dropped by an average of 16% per year since the introduction of the SDRC into NLCP. SDRC seems then able to reduce leprosy detection over the years following its administration by interrupting transmission.

With regard to examined trends according to epidemiological characteristics of cases, leprosy detection was decreasing from 2000 to 2017 in the 12 regions of Morocco even in male, female, adult, children, urban and rural cases, all clinical forms and in cases with disabilities grade 0 and 1. The decreasing trend of leprosy was more pronounced in the second period after joinpoints. Years corresponding to joipoints were different following the characteristics but were all over 2006. With regard to grade 2 disabilities trend of leprosy detection was increasing between 2000 and 2006 followed by an inversion of the curve and a significant decreased in leprosy detection after 2006. One fact may explain the pronounced drop of detection rate in the second periods: Morocco has reviewed its control strategy for leprosy in 2006 by adopting the WHO short protocol of multidrug therapy. This protocol is more effective, suppresses infection and facilitates observance. The new organization of NLCP after 2006 was accompanied as well by a decentralization of leprosy health care allowing better geographical access to services for rural areas, early case detection, early treatment and closed contact surveillance; leading then to the reduction of new case detection in the country. To our knowledge, national reporting protocols and trends have remained constant and rigorous over the period studied.

Stratified analysis showed that leprosy detection rate decreased significantly prior to 2012 intervention in female cases, adults and in paucibacillary forms. These results are common findings in countries with declining leprosy like Morocco, as there is evidence of decreases in female to male sex-ratio and in paucibacillary forms among new cases. The proportion of paucibacillary cases has been found to decrease in several populations concomitant with declining incidence [15]. These patterns probably reflect an increase in the proportion of long incubation period cases as the disease disappears. Long incubation periods are associated more with multibacillary than with paucibacillary forms, and multibacillary cases are associated with males [16]. In the same way, if MDT affects the transmission of infection it should also reduce the detection rate of paucibacillary cases first because of their shorter incubation periods. Our findings can be then the results of MDT and decline in transmission. Moreover, differences in sex-ratios in low endemic countries can be influenced by susceptibility to infection or to disease and also by potential bias in case ascertainment. Social stigma and sociological patterns that confine females to houses in some settings may be related to low case reporting of females. On the other hand, 94% of leprosy cases registered in the study period were adult forms, those cases significantly decreases after 2012. This can be due to general effect of SDRC.

The present study is the first one that reported data on leprosy detection in Morocco in the last two decades. It has the strength to gather information comprising all cases reported nationally through NLCP; however, some limitations of the study have to be considered when interpreting our findings: 1/ Data of NLCP may present inconsistencies in the quantity and quality of information over time and between the regions. Case detection maybe under-reported, despite the progress achieved during the observational period in terms of active case findings (examination of household contacts), early MDT treatment (WHO protocols), social support, training of health care workers, integration of NLCP activities into primary healthcare services and implementation by general practitioners. 2/ Stratified analysis showed that leprosy declined significantly prior to 2012 intervention in female cases, adults and in paucibacillary forms. Other possible factors related to 2006 policy changes in NLCP may also contribute to the decreasing trend of leprosy in the country. 3/ There is no doubt that NLCP control measures have played an important role in leprosy decline; however, improvements in socioeconomic conditions over the country, implementation of laws reducing stigma and other cultural factors should also be considered, even if their contribution to leprosy decline remains difficult to assess. These issues deserve further investigations and may provide valuable insights. 4/ Stratified analysis by regions have showed a decrease in leprosy detection. The decreasing trend of leprosy after joinpoints was not significant in all regions except for two ones (Béni-Mellal-Khénifra and Marrakech-Safi). This can be due to a less of statistical power of analysis because of limited recoil of the study (only 5 years after the joinpoint). 5/ The joinpoint regression analysis used in the study is an analytical but exploratory method that does not take into account factors that may influence leprosy trends (SDRC, level of regional implementation of NLCP, etc). A further study, using interrupted time series analysis with a multivariate approach is required and is currently on the way in order to assess, in statistical terms, how much an intervention changed an outcome of interest and whether factors other than the intervention could explain the change [17]. Despite these limitations, the data analyzed in our study are consistent and representative for a country over a period of 17 years.

In conclusion, Morocco’ decline in leprosy reflects a global trend. This time series demonstrated a drop in case detection by 16% per year since SDRC implementation. This fact can allow us generating a hypothesis about a possible influence of SDRC on accelerating the reduction of leprosy detection in Morocco. An interrupted time series analysis is needed to confirm this hypothesis.

## Supporting information

S1 STROBE statement Checklist of items that should be included in reports of observational studies. (DOCX)

## References

[1] WHO. Global leprosy update, 2015: time for action, accountability and inclusion. *Weekly epidemiological record*. 2016; 91: 405–420. 27592500

[2] Reveiz L, Buendía JA, Téllez D. Chemoprophylaxis in contacts of patients with leprosy: systematic review and meta-analysis. *Rev Panam Salud Publica*. 2009; 26: 341–349. 20107683 10.1590/s1020-49892009001000009

[3] Richardus JH, Oskam L. Protecting people against leprosy: chemoprophylaxis and immunoprophylaxis. *Clin Dermatol*. 2015; 33: 19–25. 10.1016/j.clindermatol.2014.07.009 25432807

[4] Moet FJ, Pahan D, Oskam L, Richardus JH, COLEP Study Group. Effectiveness of single dose rifampicin in preventing leprosy in close contacts of patients with newly diagnosed leprosy: cluster randomised controlled trial. *BMJ*. 2008; 336:761–764. 10.1136/bmj.39500.885752.BE 18332051 PMC2287265

[5] Ferreira SMB, Yonekura T, Ignotti E, Oliveira LB, Takahashi J, Soares CB. Effectiveness of rifampicin chemoprophylaxis in preventing leprosy in patient contacts: a systematic review of quantitative and qualitative evidence. *JBI Database System Rev Implement Rep*. 2017; 15: 2555–2584. 10.11124/JBISRIR-2016-003301 29035966

[6] Feenstra SG, Pahan D, Moet FJ, Oskam L, Richardus JH. Patient-related factors predicting the effectiveness of rifampicin chemoprophylaxis in contacts: 6 year follow up of the COLEP cohort in Bangladesh. *Lepr Rev*. 2012; 83: 292–304. 23356030

[7] WHO. World Health Assembly resolution to eliminate leprosy as a public health problem by year 2000 World Health Organisation, Geneva, 1991.

[8] Programme National de Lutte contre la Lèpre. Guide de la lutte anti-lépreuse à l’usage des professionnels de santé Ministère de la Santé, Maroc, 2014.

[9] Kim HJ, Fay MP, Feuer EJ, Midthune DN. Permutation tests for joinpoint regression with applications to cancer rates. *Stat Med*. 2000; 19: 335–351. 10649300 10.1002/(sici)1097-0258(20000215)19:3<335::aid-sim336>3.0.co;2-z

[10] Wilmot KA, O'Flaherty M, Capewell S, Ford ES, Vaccarino V. Coronary Heart Disease Mortality Declines in the United States From 1979 Through 2011: Evidence for Stagnation in Young Adults, Especially Women. *Circulation*. 2015; 132: 997–1002. 10.1161/CIRCULATIONAHA.115.015293 26302759 PMC4828724

[11] Joinpoint Regression Program, Version 4.2.0.2—June 2017; Statistical Methodology and Applications Branch, Surveillance Research Program, National Cancer Institute.

[12] Moet FJ, Pahan D, Oskam L, Richardus JH; COLEP Study Group. Effectiveness of single dose rifampicin in preventing leprosy in close contacts of patients with newly diagnosed leprosy: cluster randomised controlled trial. *BMJ*. 2008; 336: 761–764. 10.1136/bmj.39500.885752.BE 18332051 PMC2287265

[13] Bakker MI, Hatta M, Kwenang A, Van Benthem BH, Van Beers SM, Klatser PR, Oskam L. Prevention of leprosy using rifampicin as chemoprophylaxis. *Am J Trop Med Hyg*. 2005; 72: 443–448. 15827283

[14] Idema WJ, Majer IM, Pahan D, Oskam L, Polinder S, Richardus JH. Cost-effectiveness of a chemoprophylactic intervention with single dose rifampicin in contacts of new leprosy patients. *PLoS Negl Trop Dis*. 2010; 4: e874 10.1371/journal.pntd.0000874 21072235 PMC2970532

[15] Irgens LM, Skjaerven R. Secular trends in age at onset, sex ratio and type index in leprosy observed during declining incidence rates. *Am J Epidemiol*.1985, 122: 695–705. 3875282 10.1093/oxfordjournals.aje.a114148

[16] Koba A; Ishii N, Mori S, Fine PE. The decline of leprosy in Japan: patterns and trends 1964–2008. *Lepr Rev*. 2009; 80: 432–440. 20306642

[17] Wagner AK, Soumerai SB, Zhang F, Ross-Degnan D. Segmented regression analysis of interrupted time series studies in medication use research. *J Clin Pharm Ther*. 2002; 27: 299–309. 12174032 10.1046/j.1365-2710.2002.00430.x

